# American Medical Association

**Published:** 1891-11

**Authors:** 


### American Medical Association.

### Section of Oral and Dental Surgery.

### THE PRESIDENT'S ADDRESS.

GENTLEMEN:—Dentistry from the earliest period up to the present time has been practiced by medically educated men.

Hippocrates, 460 B. C., speaks of dentrifices and the fixing of the teeth; Celsus, at the end of the first century; Ætius, in the sixth; Egenolff and Ambrose Pare, in the fourteenth. All mention certain forms of treatment of the teeth. John Hunter, 1728, spent much of his time in the study and treatment of the teeth, as well as the study of general anatomy; Fouchard, 1747; Bourdet, 1786; Fox, 1814; Catalan, 1826, all practiced dentistry and also wrote extensively on dental subjects.

The instruments for dental as well as surgical purposes which are to be seen in the museums of Europe, together with the beautiful specimens of Etruscan and Phœnician dentistry now in the possession of Drs. Van Marter of Rome, Barrett of Buffalo, and Taft of Cincinnati, and which are similar to those made today, are striking illustrations of the superior ability which men of early times acquired.

About 1826-1830, the practice of dentistry was not wholly in the hands of an enviable class of individuals. Many were watchmakers, barbers and tinkers of various descriptions, who had taken up the practice for the money that was in it, and were roaming about the country extracting teeth and inserting artificial ones, regardless of honor and ability.

There were, however, a few medical men practicing dentistry, who had obtained a high standing in their chosen profession, and who were anxious to hold their special calling upon an equal footing with other branches of the healing art. Among those, whose names stand out conspicuously in the history of the country, are, Parmley, Brown, Hayden, Tucker, Hudson, Greenwood, Maynard, Trenon, Harwood, Chapin A. Harris and Keep.

We can easily imagine the feeling of these men, who by reason of superior skill and ability had attained high positions in their chosen specialties, when they met in council together to discuss the different subjects relating to the standing of the calling with other specialties, and the qualifications for admission to their Society. It was not until 1839 that any movement was made on the part of these men to elevate their specialty from the slum in which a majority of the so-called practitioners were holding it. It was at this period that one of the most important events in the history of dentistry occurred.

The *American Journal of Dental Science* made its first appearance, with Dr. Chapin A. Harris and Eleazer Parmley as its editors. The American Society of Dental Surgery was organized in 1840, also through his efforts in co-operation. In connection with these movements, it was the ambition of Dr. Harris to organize a dental school in connection with the medical department of the University of Maryland. The practice of dentistry (at this time), with few exceptions, being at a very low ebb, did not impress the faculty of the university as being of sufficient importance to be considered a part of the healing art. The request of Dr. Harris was therefore rejected. Whether this request, at this period in the history of dentisty, would have been rejected by the faculty of any other medical college in this country, or whether it would have been rejected if presented to the medical department by any other person, or at any other time, we have no means of knowing. Nor do we know much of the general feeling among physicians at that time, in regard to the relation of dentistry to medicine. We only know that the rejection of the proposition gave Dr. Harris new energy, and as a result the Baltimore College of Dental Surgery was established, and that the School of Medicine and the School of Dentistry ignored each other for a time.

Whether the establishment of a strictly dental school, together with the conferring of a separate degree from that of the medical school, has benefited dentistry, it is difficult to say.

The original idea of Dr. Harris, if it could have been successfully carried out, would no doubt have placed dentistry as a calling upon a higher plane than it occupies at the present time. His idea was that by this union the dental department, being a legitimate specialty of medicine, would thus draw support from the medical profession at large. Be that as it may, dentistry, forced upon its career with a new degree, had, with very little aid from the medical profession, to work its own way as best it could. This incident marked one of the greatest epochs in the history of our calling.

Like the young man, sent away from the parental roof, dentistry has grown strong in some of its features. The Baltimore College, owing to the talent of unexceptionally able men, flourished, and men of ability were graduated, who practiced dentistry in different parts of the country, with the new degree of "Doctor of Dental Surgery." So successful was this college, that other schools of dentistry were organized shortly afterward, and these in their turn became as successful as the mother school.

From the first, graduates of dentistry who were ambitious to excel have never been satisfied with a dental degree only, and many men, anxious to acquire higher attainments, have taken the medical degree also. Many of the graduates are anxious to acquire a broader and more liberal education, but time and purse do not permit it.

Dental students have been ambitious to obtain the highest acquirements, and, in many cases, have not been satisfied with the course of instruction in our dental colleges. The dental faculties have been obliged to so shape their course, and so far as possible have made arrangements with medical colleges, that by taking one additional course, their students could receive the medical as well as the dental degree. And there has been a desire on the part of both medical and dental graduates to draw the medical and dental schools together, as evidenced by the fact that medical men are anxious to secure positions in the dental colleges. This desire may not have been outwardly intentional on the part of some, but the relations of one to the other are so close that scarcely any distinction really exists. I have frequently heard medical men, those who have had experience as teachers in both colleges, say that they prefer to lecture to dental students.

The first dental college to unite with a medical college was the St. Louis college. The St. Louis Dental College was founded in September, 1866, and the first announcement stated that the students will have the benefit of the lectures in anatomy, chemistry, physiology, materia medica that are given to the matriculants of the St. Louis Medical College, the first college to take the step in this direction.

Following this, in 1867, the Harvard Dental School was organized in connection with Harvard University; since that date most of the universities in this country have established dental departments. The older dental colleges are uniting themselves as rapidly as possible to universities, so that at the present time nearly one-half the dental colleges are departments in the different universities. Ever since the formation of the Massachusetts Medical Society, in 1781, it has had members practicing dentistry; and the Suffolk District Medical Society, which is a part of the Massachusetts State Medical Society, created a section in 1866 called No. 5, for "Surgery and Dentistry;" this is now a quarter of a century old. In Massachusetts, forty or fifty years ago, no reputable dentist would take a pupil, unless he would engage to take a full medical course, or had already graduated in medicine. Scarcely a dentist who has been in practice for twenty-five years. and who has sons growing up, is not anxious to have them to take a full course in medicine before taking up the subject of dentistry, which they regard as a specialty in medicine.

This brief history has been given to show that our best educated early practitioners in dental surgery have always regarded it as a specialty in medicine rather than an independent profession. Dr. Harris so regarded it, and he was the founder of our first dental college, and established this college for the reason that he was not permitted to organize a dental department in a medical college, as he preferred to do. This is evidenced from his inaugural address at that time.

"Allow me," he said, "to observe, however much of interest or curiosity the establishment of this institution may have awakened, it constitutes an era in the history of a most useful and valuable department of medicine."

In speaking of the empiricism that had up to that time existed in the practice of dentistry, he says: "I feel bound to the public and to my own reputation, to denounce the empiricisms that have, and do still exist in the department of medicine." Again he says: "It is to be hoped that the day is not far remote when it will be required of those to whom this department of surgery is intrusted, that they shall be educated men."

Although dentistry apparently seceded from the mother profession in 1840, it was so near akin to it, that with few exceptions, there has been a yearning on the part of the dental graduates to return to the parental roof. Many graduates of dentistry have availed themselves of the opportunity, and the numbers are increasing every year.

With the convening of this meeting, the Section of Dental and Oral Surgery in the American Medical Association enters upon the tenth year of its existence. At the session of the American Medical Association held in Richmond, Va., 1881, Drs. W. W. Allport, J. W. Brophy, E. S. Talbot, Chicago; Dr. J. L. Williams, Boston; Dr. G. L. Goodwillie, New York; Dr. Hauxhurst, Grand Rapids, Mich.; and Dr. G. L. Parmlee, Hartford, Conn., practitioners of dentistry, holding medical degrees, presented themselves as delegates from local medical societies, for the purpose of organizing a Section on Dental and Oral Surgery. Each gentleman constituted himself a committee of one to champion the movement among the members of the Association who were in attendance. They found no opposition whatever; on the contrary, the oldest members, and especially the ex-Presidents of the Association, were heartily in sympathy with the movement, and were anxious to assist in bringing it about. At the morning session Thursday, May 5, Dr. Samuel D. Gross of Philadelphia, asked for a suspension of the regular order of business, which motion was granted. He then moved that the By-laws of the Association be so amended as to create another Section, to be known as No. 7, entitled Dentistry. The motion was favored by Dr. Sayre, of New York, and Dr. N. S. Davis, of Chicago, and was adopted. The object of suspending the rules at this time, was for the purpose of creating the section, so that we might organize and commence work at that session; the members who were to constitute the Section being prepared with papers for that purpose. Dr. Toner, of Washington, objected, saying that he was not opposed to the Section organizing and commencing work this year, but he did not wish to make a precedent for future Sections. It was therefore decided that it could not go into operation until the next year, when, according to the Constitution and By-laws of the Association, dentistry was officially recognized by the American Medical Association.

The effect that this movement produced upon the so-called dental profession was, in one respect, magical. Before this period, scarcely a meeting of dentists convened, without having upon its programme a paper upon the subject of "Is Dentistry a Specialty in Medicine?" Strange as it may seem, many took the ground that it was not, ignoring the fact that it had been practiced as such until, and by some within, the last forty years; that many of its branches were taught by medically educated men, and also that we were practicing on a part of the human body. Since this period, scarcely a paper has been written upon this subject. The action taken by the American Medical Association, as well as cases which have been lately decided by the courts, have legally settled the question forever.

Fortunately, there is little opportunity for wire-pulling or scheming for political preferment in our Section, for it is well known that the majority of our members prefer that the offices be given to others rather than to themselves. Thus the time devoted to the Section has been entirely given up to the reading and discussion of papers, and in no dental society in this country, has there been presented such an array of scientific papers as has been given in this Section. The men who have taken part in the meetings of this Section have in most cases been of exceptional ability, and whose standing in the specialty has added dignity to its meetings. The influence of the work in this Section upon the medical profession has brought about a marked change in the Association.

The Section of Dental and Oral Surgery is recognized as a part of the whole, like other Sections, and its members exert as much power in promoting the welfare of the body, as do the members of other Sections. Indeed, the members of this Section have had the pleasure of listening to papers and discussions at its sessions, by some of the ablest men of the Association, which not only added interest to its meetings, but also showed that the members of the Association are in full accord with the specialty as a part of the general body.

Since the organization of the Section, editors of medical journals have become quite liberal, as well as more intelligent, in their discussion of dental subjects, and have taken more interest generally in the affairs of dentists; and physicians have accepted invitations to read papers before local dental societies, and dentists have read papers before medical societies. This shows the interest which an interchange of thought is developing, by the union of the different branches of the healing art. Since the formation of this Section, through the efforts of W. W. Allport, teaching in regard to dental diseases has been established in many of the medical colleges of Chicago, which example has been followed in other colleges of the country. One of the arguments used by some dentists against the theory that dentistry is a specialty in medicine, is that medically educated persons know nothing of dentistry, or those diseases of the oral cavity which result from diseased teeth. Medical men could hardly be expected to know much in regard to the lesions of the mouth, they having received no special instruction upon this subject. The action taken by Dr. Harris and the medical faculty in 1840, no doubt impressed the faculties of other medical colleges with the idea, that lesions of the mouth resulting from diseased teeth were of little consequence, and therefore knowledge of them of little value, to the general practitioner.

Now however, medical men, as well as dentists, know that many lesions of the body are the direct outcome of diseased teeth. In my capacity as a dental teacher in a medical college, I have instructed the students for the past eight years that (in my opinion) many of the diseases of the body, such as pneumonia, consumption, typhus and typhoid fever, eruptive fever, suppuration of the throat and tonsils, aphthæ, ulcers, etc., were the outcome of a collection of micro-organisms in the mouth, and decay of the teeth. My reasons for this theory were that, in many instances in my practice, I have observed patients entirely recover from the supposed consumption and other bodily ailments, after the mouth has been put in a healthy condition. In one case a lady gained 35 lbs. in weight, a young man gained 13 lbs., and a young girl who had been treated for six months for consumption, and was supposed to be on her death-bed, entirely recovered after having a number of roots of teeth removed and tonics administered. This theory has been confirmed by Professor Miller, of Berlin, who has observed the bacillus tuberculosis and the bacilli of other diseases in the mouth. In his work upon "Micro-organisms of the Human Mouth," he shows how many diseases of the body are produced by pathogenic bacteria by inspiration, absorption, and being taken into the alimentary canal, which have accumulated in the mouth. To-day many of the medical colleges of the country have a chair upon dental and oral surgery, and the students are now taught dental anatomy, physiology and pathology. Of the six medical colleges in Chicago, there is not one that does not provide for instruction in these branches by some dentist. No medical student should be allowed to graduate without some knowledge of the laws of diseases, and their effects on the mouth and teeth, and no medical college to-day can be considered complete without a chair upon this subject. In view of the fact that able practitioners of dentistry were debarred from becoming members of the American Medical Association, on the ground that they did not belong to some local medical society, at a meeting held in Chicago, in June, 1887, Dr. Allport conceived the idea of having a resolution passed, which should admit as members men of ability who held the D.D.S. degree.

With the assistance of Dr. N. S. Davis, the following resolution was presented and unanimously adopted by the American Medical Association, 1887:

*Resolved*, That the regular graduates of such dental and oral schools and colleges as require of their students a standard of preliminary or general education, and a term of professional study, equal to the best class of the medical colleges of this country, and embrace in their curriculum all the fundamental branches of medicine, differing by substituting practical and clinical ins struction in dental and oral medicine and surgery, be recognized as members of the regular profession of medicine, and eligible to membership in this Association, on the same conditions and subject to the same regulations as other members.

In the following year, 1888, the Chicago Dental Club, having adopted the Code of Ethics of the American Medical Ascociation, sent the following members to the Association, as qualified under the resolution: W. W. Allport, A. E. Baldwin, John Marshall and E. S. Talbot; and since that year, 1888, the Dental Club has continued to send members to the Association. The adoption of this resolution by the representative society of the country, would seem to show that the medical profession had done its full part to recognize *properly educated* dentists as legitimate specialists in the practice of medicine. The question of relationship has now been definitely settled forever, and the only question now remaining is this: What proportion of dentists, in the future, will so qualify themselves to practice dental and oral surgery, that they will have the right to be classed as medical specialists, the same as surgeons and ophthalmologists, and entitled to recognition in the Association, in accordance with the letter and spirit of the resolution referred to?

Professor Garretson, in a letter to me, states that in the Philadelphia Dental College, which is in connection with the Medico Chirurgical College, the following distinction is made between the course conferring the D.D.S. degree and the one conferring the M.D. degree, or both: On matriculation, the student signifies his intention to take the M.D., or a dental degree, or both. If he chooses the course conferring the dental degree, he is taught only such branches as pertain to the treatment and filling of teeth. On the other hand, if he signifies his intention to take the M.D. degree, or both, he receives instruction in the branches that will fit him to practice oral surgery, or oristry, and medicine, as well as dental surgery. The professional position that the educated dentist occupies at the present time, could not be improved upon, as is true of the position he holds in society. His relations with the mother profession are as free and broad as the air of this great American continent. It now remains for him to decide whether he will be satisfied with little education—with an education that permits him to see only faintly, and to realize not at all, the possibilities of his profession, and renders him content to grope along in the lower stratum of his practice, seeing and wishing nothing higher, possessing only narrow views in regard to his calling, attending no societies, or only those whose time is given to discussions on such subjects as red rubber, amalgam and root filling, extraction of teeth and insertion of artificial dentures. If he is content with these things, and looks upon the profession of dentistry as a trade, merely as a means of subsistence, with as little expenditure of power and thought from himself as possible; will he rest satisfied with this position, or will he take a broader view of the situation, looking at dentistry from all sides, thinking the subject worthy of all his power, all his talents and ability, striving to make it equal to the mother profession, and educating himself so that he may stand upon an equal footing, shoulder to shoulder with the best medical men in the country.

Some one may say that he has not the time nor the money for this. In answer to this, permit me to refer to a Professor in the University of Berlin, to show that it is possible for a poor American boy to *educate* himself for the practice of dentistry.

His worth was recognized by the faculty of the University of Berlin, and a position was offered him as Professor in the University, an honor that has been accorded to no other American. And during my late visit to Berlin, it gave me great pleasure to observe the respect shown by the faculty of the University, Government officials, as well as the highest officer in the International Medical Congress, to Professor W. D. Miller. This example is only given to show that to him who works in the right direction, all things are possible, and the higher he places the limits of his attainments, the greater will his attainment be.

Let the dentist be a scientist, and not a mediocre and narrow-minded tradesman. Let him select a new mark every day, at which to aim his energies and talents, and let that mark be ever ahead, and ever on a higher plane.

